# Applying the WHO ICD-PM classification system to stillbirths in a major referral Centre in Northeast Nigeria: a retrospective analysis from 2010-2018

**DOI:** 10.1186/s12884-020-03059-8

**Published:** 2020-07-01

**Authors:** Eseoghene Dase, Oghenebrume Wariri, Egwu Onuwabuchi, Jacob A. K. Alhassan, Iliya Jalo, Nazeem Muhajarine, Uduak Okomo, Aliyu U. ElNafaty

**Affiliations:** 1 Department of Obstetrics and Gynaecology, Federal Teaching Hospital Gombe, Gombe, Nigeria; 2 African Population and Health Policy Initiative, Gombe, Gombe State Nigeria; 3 Vaccines and Immunity Theme, Medical Research Council (MRC) Unit The Gambia at The London School of Hygiene and Tropical Medicine, Banjul, The Gambia; 4 Aberdeen Centre for Health Data Science (ACHDS), Institute of Applied Health Sciences, University of Aberdeen, Aberdeen, UK; 5 Department of Community Health and Epidemiology, College of Medicine, University of Saskatchewan, Saskatoon, Canada; 6 Department of Paediatrics, Federal Teaching Hospital Gombe, Gombe, Nigeria; 7 Department of Paediatrics, College of Medical Sciences, Gombe State University, Gombe, Nigeria; 8 Department of Obstetrics and Gynaecology, College of Medical Sciences, Gombe State University, Gombe, Nigeria

**Keywords:** Stillbirths, WHO ICD-PM, Gombe, Nigeria

## Abstract

**Background:**

Lack of a unified and comparable classification system to unravel the underlying causes of stillbirth hampers the development and implementation of targeted interventions to reduce the unacceptably high stillbirth rates (SBR) in sub-Saharan Africa. Our aim was to track the SBR and the predominant maternal and fetal causes of stillbirths using the WHO ICD-PM Classification system.

**Methods:**

This was a retrospective observational study in a major referral centre in northeast Nigeria between 2010 and 2018. Specialist Obstetricians and Gynaecologists assigned causes of stillbirths after an extensive audit of available stillbirths’ records. Cause of death was assigned via consensus using the ICD-PM classification system.

**Results:**

There were 21,462 births between 1 January 2010 and 31 December 2018 in our study setting; of these, 1177 culminated in stillbirths with a total hospital SBR of 55 per 1000 births (95% CI: 52, 58). There were two peaks of stillbirths in 2012 [62 per 1000 births (95% CI: 53, 71)], and 2015 [65 per 1000 births (95% CI, 55, 76)]. Antepartum and intrapartum stillbirths were almost equally prevalent (48% vs 52%). Maternal medical and surgical conditions (M4) were the commonest (69.3%) cause of antepartum stillbirths while complications of placenta, cord and membranes (M3) accounted for the majority (45.8%) of intrapartum stillbirths and the trends were similar between 2010 and 2018. Antepartum and intrapartum fetal causes of stillbirths were mainly due to prematurity which is a disorder of fetal growth (A5 and I6).

**Conclusions:**

Most causes of stillbirths in our setting are due to preventable causes and the trends have remained unabated between 2010 and 2018. Progress toward global SBR targets are off-track, requiring more interventions to halt and reduce the high SBR.

## Background

Globally, an estimated 2.6 million babies are stillborn (die in the last 3 months of pregnancy or during childbirth) with a Stillbirth rate (SBR) of 18.4 per 1000 births [1]. The overwhelming majority of these stillbirths occur in low- and middle-income countries (LMICS), particularly in Sub Saharan Africa and South Asia [2], and can be prevented through equitable and high-quality coverage of care for all women and newborns. Based on the *Every Newborn* Action Plan to improve newborn health and prevent stillbirths, a stillbirth target of 12 or less stillbirths per 1000 total births for all countries by 2030 was set, with a focus on addressing inequalities and the use of audit data to track and prevent stillbirths [3]. However, implementation will require that countries strengthen their ability to collect, compile, analyse, and report on data from the period of commencement, as well as maintain robust databases with constant data revolution [4].

In 2015, Nigeria was estimated to have the second highest stillbirth rate in the world (42.9/1000 births) [2]. National progress towards ending preventable stillbirths has been slow (annual rate of reduction approximately 1.3%) in the context of weak health systems with limited infrastructure and populations faced with high out-of-pocket expenditures, poverty, inequality and societal unrest [2, 5]. Complex humanitarian emergencies resulting from conflict in certain parts of the country have resulted in dramatic movements of people (including pregnant women and newborns), compromising access to health care [6]. This is most evident in northeast Nigeria where religious extremism and Boko Haram insurgency over the last decade have led to destruction of public utilities and socio-economic infrastructure with displacement of many communities and inequitable access to quality health services for vulnerable populations, notably women and children [6]. The direct consequences of this are marked regional disparities in coverage of essential health interventions and poor data reporting, thereby masking the true burden of maternal, newborn and child deaths including stillbirths.

Data on the number of deaths or the causes is essential to improve the quality of care, prevent future deaths, allocate resources, improve vital statistics, and reach global targets [7]. Registration of stillbirths and newborn deaths should be accompanied by programmatically relevant categorisation of the causes of death. The World Health Organization (WHO) recently adapted the International Classification of Diseases (ICD-10) for use in perinatal mortality (ICD-PM) [8]. The ICD-PM provides a standardised system for classifying perinatal mortality (including stillbirths) based on time of death (antepartum or intrapartum) into fetal and maternal causes thereby enabling comparisons within and between diverse settings and contexts [8]. The ICD-PM has been shown to work well using stillbirth data from the United Kingdom and South Africa [9, 10] and more recently in a multi-country setting in Kenya, Malawi, Sierra Leone and Zimbabwe [11]. To date, there is no such study that has used the ICD-PM system to classify stillbirths from Nigeria. We, therefore, applied the WHO ICD-PM retrospectively to determine the trends in SBR between 2010 and 2018 and the causes of stillbirths in a major tertiary referral health facility in northeast Nigeria.

## Methods

### Study setting

**Fig. 1:**
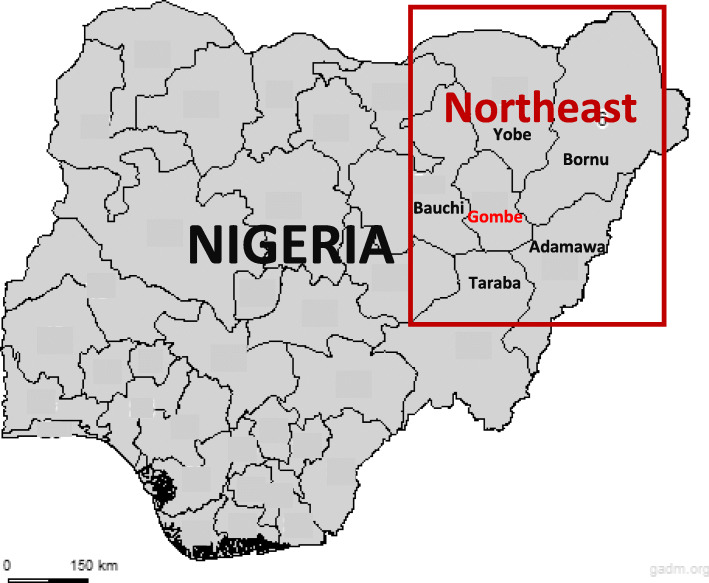
Map of Nigeria showing northeast region and study area (Gombe State). Map source: adapted by authors based on data from GADM maps and data (https://gadm.org/index.html). GADM maps and data are freely available for academic and other non-commercial use

This was a retrospective study conducted at the Federal Teaching Hospital, Gombe (FTHG), in Gombe State, northeast Nigeria. Centrally located in this region, it shares borders with five other states - Adamawa, Bauchi, Bornu, Taraba, and Yobe (Fig. 1). Unlike its neighbouring states, Gombe is one of the states least affected by the armed conflict and insurgency in northeast Nigeria [12]. The estimated population of 3.3 million fluctuates due to movement of internally displaced persons from other states in the region [13]. The 450-bed capacity FTHG is a tertiary referral facility, receives patients from all five surrounding states. The hospital provides Comprehensive Obstetric care and is staffed with all cadres of the obstetric health workforce, including obstetricians/gynaecologists and midwives as well as anaesthetists and neonatologists. The average annual delivery rate for the hospital is about 2400 deliveries and the caesarean section rate is 27%.

### Data collection

We identified all stillbirths recorded in the hospital between 1st January 2010 and 31st December 2018 from delivery room, obstetric ward and theatre registers. Since stillbirth data are routinely documented in the maternal admission notes, we therefore retrieved the antenatal and admission records of mothers who had delivered a stillborn baby during the period under review. Using a pre-tested data extraction form, we collected the following data: maternal characteristics (age, parity, ethnicity and occupation); obstetric factors (antenatal attendance, gestational age at delivery, prior stillbirths, mode of delivery, obstetric and medical conditions); and fetal factors (birth weight, sex, congenital anomalies and type of stillbirth).

### Definition of terms and variables

#### Stillbirth

For international comparison, stillbirth was defined as any fetal death occurring prior to the complete expulsion of the fetus at *≥*28 weeks of gestation (third trimester deaths) or with birth weight of *≥*1000 g [14].

#### Fresh stillbirths (FSB)

Stillbirths in which the fetus was expelled with an intact skin suggesting that the death occurred during labour (Intrapartum) or less than 8 h before delivery. The categorisation as FSB was done by the attending doctors and nurses at the time of delivery.

#### Macerated stillbirth (MSB)

Any fetus expelled with signs of skin degeneration, suggesting the death have occurred before labour (antepartum) or more than 8 h before expulsion. The categorisation as MSB was done by the attending doctors and nurses at the time of delivery.

#### Antepartum death

In this study, antepartum death was defined as stillbirths whose mothers arrived at the healthcare facility before the onset of labour with absent fetal heart sound on examination or the delivery of a macerated stillbirth where the time of fetal demise is not known.

#### Intrapartum death

In this study, intrapartum death was defined as death of the fetus during labour in which the mother arrived with fetal heart sounds present and or the delivery of a fresh stillbirth where the time of fetal death is unknown.

#### Booked pregnancy

A booked pregnancy was defined as any pregnancy that was registered and or received antenatal care (ANC) in FTHG (as evidenced by the antenatal records) irrespective of the number of such ANC visits.

#### Unbooked pregnancy

This was any pregnancy that did not receive ANC at the FTHG but presented only during labour. Women who received ANC elsewhere were also classified as unbooked because their antenatal records were not available to the attending obstetrics team.

#### Gestational age

This was categorised according to the gestational weeks at delivery. This included; preterm (28- < 37 weeks), term (37–42 weeks) and post term which was > 42 weeks at delivery [15].

#### Birth weight

This was the measured weight of the stillborn at delivery. This was categorised into; Extreme low birth weight (< 1000 g), very low birth weight (1000–1499), low birth weight (1500-2499 g) normal birth weight (2500-3999 g) and macrosomia which was birth weight ≥ 4000 g at delivery [16, 17].

### Application of ICD- PM classification system and assignment of cause of death

The ICD 10-PM system classifies fetal causes of stillbirths based on the time of death as either Antepartum (designated as codes with prefix **A**) or Intrapartum (codes with prefix **I**), while maternal causes of perinatal mortality (including but not limited to stillbirths) are coded with the prefix M (Supplementary Table 1) [8]. The determination of the likely cause of death and classification of the stillbirth into the ICD-PM sub-categories was independently carried out by two of the authors, ED and OE based on data from the maternal records. The final ICD-PM categories were arrived at by consensus and in cases where consensus is not reached, a third author was consulted. Due to the challenges with accurate dating of pregnancies, birth weight was used as a surrogate for gestational age as follows: fetuses < 2500 g were coded under prematurity/ low birth weight/ growth restriction (A5/I6) [8, 18]. However, prematurity/ low birth weight was only assigned as the cause of stillbirth when no other fetal cause was identified after an extensive review of the case and application of the ICD-PM tool.

### Data analysis

All statistical analyses were performed with Statistical Package for the Social Sciences (SPSS) (IBM, NY, version 24). The main outcome measures were the hospital-based Stillbirth Rate (SBR, stillbirths per 1000 births) and disaggregated causes of stillbirth based on ICD-PM classification system. We report SBR with accompanying 95% confidence intervals (CI). Categorical and continuous variables are summarised respectively as proportions and means (standard deviations). Cross-tabulations with the outcome were performed using the χ^2^ statistic for categorical variables, with statistical significance defined as alpha < 0.05 (two-sided).

## Results

**Fig. 2:**
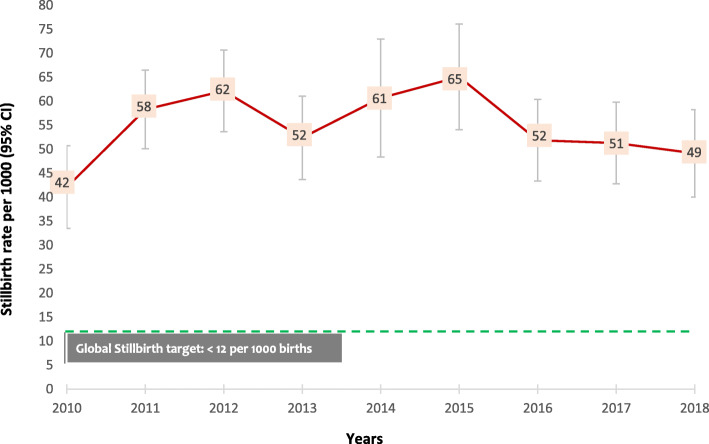
Trends in stillbirth rate per 1000 births at the Federal Teaching Hospital, Gombe, Nigeria, 2010–2018

Between 1 January 2010 and 31 December 2018 there were 21,462 deliveries (supplementary Table 2) at the Federal Teaching Hospital, Gombe, of which 1177 resulted in stillbirths giving an overall hospital SBR of 55 per 1000 births (95% CI: 52, 58). The annual SBR ranged between 42 per 1000 births (95% CI:34,51) and 65 per 1000 births (95% CI: 55,76) during the period under review (Fig. 2), with an average annual reduction rate of about 2%. There were two peaks in yearly stillbirths in 2012 and in 2015 (Fig. 2). Relevant maternal and fetal data needed for ICD-PM classification were available for all 760 (65%) cases of the stillbirths retrieved for the period under review, and these cases were included in the descriptive analyses. The 35% (417) of cases of stillbirths not included in the analysis were due to missing clinical notes and inability to retrieve maternal/foetal records which were all paper-based records.

### Background maternal and fetal characteristics

Table 1 describes the maternal and fetal characteristics of the stillborn babies. The mean maternal age was 27.6 years (SD: 6.6) and did not differ (*p* = 0.294) between women who had experienced an antepartum stillbirth compared to those who had experienced an intrapartum stillbirth. The majority (668/760; 87.9%) of women who experienced a stillbirth were aged 16–35 years. About four fifths (625/760; 82.2%) of stillborn babies were delivered by women who did not receive ANC.

**Table 1:** Demographic and clinical characteristics of women who experienced a stillbirth and their stillborn babies at the Federal Teaching Hospital, Gombe, Nigeria, 2010–2018

Variables	Categories	Antepartum ***n*** = 365 (%)	Intrapartum ***n*** = 395 (%)	Total ***n*** = 760 (%)	P
**Maternal Age (years)**	< 16	2 (0.5)	1 (0.3)	3 (0.4)	
	16–35	315 (86.3)	353 (89.4)	668 (87.9)	
	> 35	48 (13.2)	41 (10.4)	89 (11.7)	0.294
	Mean (SD)	27.5 (6.6)	27.2 (6.7)	27.6 (6.6)	0.731
P**arity**	Nullipara (0)	118 (32.1)	88 (22.3)	205 (27.0)	
	Primipara (1)	37 (10.2)	47 (11.9)	84 (11.1)	
	Multipara (2–4)	106 (29.1)	120 (30.4)	226 (29.8)	
	Grand multipara (≥5)	104 (28.6)	140 (35.4)	244 (32.1)	0.004
**Antenatal care**	No ANC	280 (76.7)	345 (87.3)	625 (82.2)	
	1–3 visits	54 (14.8)	29 (7.4)	83 (10.9)	
	4–7 visits	30 (8.2)	17 (4.3)	47 (6.2)	
	≥8	1 (0.3)	4 (1.0)	5 (0.7)	0.001
**Type of pregnancy**	Singleton	352 (96.4)	369 (93.4)	720 (94.9)	
	Multiple	13 (3.6)	26 (6.6)	39 (5.1)	0.050
**Mode of delivery**	Vaginal^a^	306 (83.8)	265 (67.1)	571 (75.1)	
	Caesarean section^b^	59 (16.2)	130 (32.9)	189 (24.9)	< 0.001
**Previous stillbirth**	No	304 (83.2)	331 (83.8)	634 (83.5)	
	Yes	61 (16.8)	64 (16.2)	125 (16.5)	0.845
**Year of stillbirth**	2010	29 (8.0)	28 (7.1)	57 (7.5)	
	2011	44 (12.1)	85 (21.5)	129 (17.0)	
	2012	65 (17.9)	60 (15.2)	125 (16.5)	
	2013	47 (12.9)	50 (12.7)	97 (12.8)	
	2014	32 (8.5)	21 (5.3)	53 (6.9)	
	2015	46 (12.6)	30 (7.6)	76 (10.0)	
	2016	37 (10.2)	38 (9.6)	75 (9.9)	
	2017	38 (10.4)	41 (10.4)	79 (10.4)	
	2018	27 (7.4)	42 (10.6)	69 (9.1)	0.489
**Sex of Stillborn babies**	Female	172 (47.1)	212 (53.7)	384 (50.5)	
	Male	193 (52.8)	183 (46.3)	376 (49.5)	0.71
**Birthweight (grams)** **of stillborn babies**	Extremely LBW (< 1000)	16 (4.4)	10 (2.5)	26 (3.4)	
	Very LBW (1000–1499)	42 (11.5)	30 (7.6)	72 (9.5)	
	Low birthweight (1500–2499)	125 (34.2)	99 (25.1)	224 (29.5)	
	Normal birthweight (2500–3999)	151 (41.4)	233 (59.0)	384 (50.5)	
	Macrosomia (> 4000)	16 (4.4)	15 (3.8)	31 (4.1)	
	∫Missing	15 (4.1)	8 (2.0)	23 (3.0)	< 0.001
	Mean (SD)	2424 (962)	2642 (842)	2540 (907)	0.001
**Duration of** **pregnancy (weeks)**	Preterm (28–36)	188 (51.5)	152 (38.5)	340 (44.7)	
	Term (37–41)	136 (37.3)	183 (46.3)	319 (42.0)	
	Post term (42)	24 (6.6)	14 (3.5)	38 (5.0)	
	∫Missing	17 (4.6)	46 (11.7)	63 (8.3)	0.001

^a^Included five (5) babies born via assisted (instrumental) vaginal deliveries

^b^Included 51 babies delivered by laparotomy because their mothers had ruptured uterus

More than half (384/760; 50.5%) of all stillborn babies weighed between 2500 and 3999 g, but the mean birthweight of the intrapartum stillbirths was significantly heavier (*p* = 0.001) than that of the antepartum stillbirths (2642 g vs 2424 g). Male and female stillborn babies were equally common (50.5% vs 49.5% respectively). Of the 760 stillbirths, 365 (48%) were antepartum deaths, while 395 (52%) were intrapartum.

### ICD-PM causes of stillbirths

Maternal medical and surgical (M4) conditions were the most common (69.3%) maternal cause of antepartum stillbirths (Table 2), while maternal complications of the placenta, cord and membranes (M1) were the most common (45.8%) maternal cause of intrapartum stillbirth. In the M4 (maternal medical and surgical conditions) ICD-PM sub-category, the major clinical conditions included hypertensive disease in pregnancy (a spectrum including chronic hypertension, pregnancy induced hypertension, preeclampsia and eclampsia) and maternal severe anaemia which accounted for 69% (218/319) and 22% (70/319) respectively (supplementary Table 3). The main clinical conditions in the M1 (complications of placenta, cord, etc.) ICD-PM sub-category included abruptio placenta [78.7% (166/211)], cord prolapse [9.5% (20/211)] and placenta previa [9% (19/211)].

**Table 2:** Application of the ICD-PM to determine causes of stillbirth at the Federal Teaching Hospital, Gombe, Nigeria, 2010–2018

	M1 Complications of placenta, cord, etc.	M2 Maternal complications of pregnancy	M3 other complications of labour & delivery	M4 Maternal medical & surgical conditions	M5 No maternal condition	Total (%)
**Antepartum Death**						
**A1**: Congenital malformations, and chromosomal abnormalities	0	7	0	2	2	**11 (3.0)**
**A2**: Infection	0	0	0	1	0	**1 (0.3)**
**A3**: Antepartum hypoxia	4	2	0	23	6	**35 (9.6)**
**A4**: Other specified antepartum disorder	0	1	0	0	0	**1 (0.3)**
**A5**: Disorder related to fetal growth	22	11	0	144	30	**206 (56.7)**
**A6**: Antepartum death of unspecified cause	4	6	0	83	17	**110 (30.1)**
**Total n = (%)**	**30 (8.2)**	**27 (7.4)**	**0 (0.0)**	**253 (69.3)**	**55 (15.1)**	**365 (100.0)**
**Intrapartum Death**						
**I1**: Congenital malformations, and chromosomal abnormalities	1	7	8	3	1	**20 (5.1)**
**I2**: Birth trauma	0	0	0	0	0	**0 (0.0)**
**I3**: Acute intrapartum event	10	3	17	8	3	**41 (10.3)**
**I4**: Infection	0	0	0	0	0	**0 (0.0)**
**I5**: Other specified intrapartum event	0	0	0	0	0	**0 (0.0)**
**I6**: Disorder related to fetal growth	87	7	26	31	5	**156 (39.5)**
**I7**: Intrapartum of unspecified cause	83	3	61	24	7	**178 (45.1)**
**Total n = (%)**	**181 (45.8)**	**20 (5.1)**	**112 (28.3)**	**66 (16.7)**	**16 (4.1)**	**395 (100.0)**
**Grand Total N = (%)**	**211 (27.8)**	**47 (6.2)**	**112 (14.7)**	**319 (41.9)**	**71 (9.4)**	**760 (100.0)**

Disorders related to fetal growth (A5), accounted for 56.7% of antenatal fetal causes of stillbirths in our study population, and were mostly associated with maternal medical (M4) conditions. Although the majority (45.1%) of intrapartum stillbirths were categorised as fetal deaths of unspecified (I7) causes, those due to disorders related to fetal growth (I6) were mainly associated with maternal complications of placenta and cord (M1).

### Trends in ICD 10-PM causes of stillbirths, 2010–2018

**Fig. 3:**
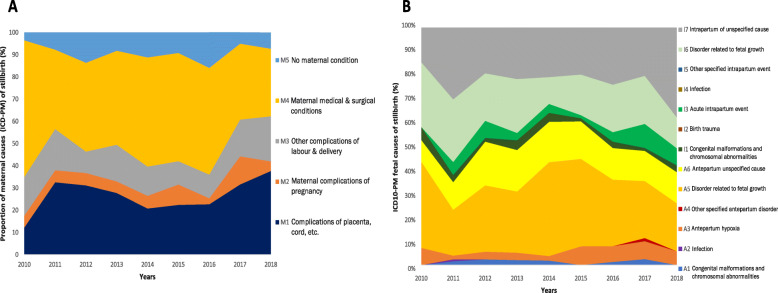
Trends in the contribution of (**a**) maternal causes and (**b**) fetal causes (ICD-PM) to stillbirths at the Federal Teaching Hospital, Gombe, Nigeria, 2010–2018

Maternal medical and surgical conditions (M4) followed by complications placenta and cord (M1) remained the highest maternal causes of stillbirth over the study period (Fig. 3a). This trend remained similar (i.e. both M1 and M4 were the most common maternal ICD-PM categories contributing to stillbirths) between 2010 and 2018, although with occasional dips. Overall, antepartum; disorder related to fetal growth (A5) and fetal death of unspecified cause (A6/I6) were the commonest fetal ICD-PM causes of stillbirth throughout, between 2010 and 2018 in our study setting (Fig. 3b).

## Discussion

We have shown a consistently high burden of stillbirths in a major urban tertiary referral hospital in northeast Nigeria over a nine-year period between 2010 and 2018, with an SBR consistently above 4 times the *ENAP* national SBR target of 12/1000 births by 2030. Based on the ICD-PM, we identified maternal medical conditions and unspecified fetal disorders as the major causes of stillbirths. Comparable facility-based data from other regions in Nigeria are lacking; however, our reported facility-based SBR is higher than the national SBR of 42.9/1000 births [19]. Sub-Saharan Africa has the highest SBR and slowest rate of progress worldwide. Nigeria and Ethiopia are the two largest contributors to this stillbirth burden [1], but our reported SBRs are much lower than that reported from northern Ethiopia (85/1000 births) [20].

Before discussing our findings further, we acknowledge the following limitations of our study. First, our inability to retrieve case records (paper-based clinical notes) for 35% of the stillbirth cases identified during the period under review. This excluded a considerable number of stillbirth cases from our analysis and may have resulted in over- or under-estimation of the different causes of stillbirths. The difficulty in accessing health records and the challenge of poor record-keeping impede the collection of better data needed to save the lives of mothers and newborns in low income settings [21]. Second, is the variability in determining maceration based on fetal visual appearance by health professionals. The shortcoming of visual examination to assess degenerative skin changes in stillborn has been documented [22]. The health care providers who classified the stillbirths into fresh or macerated categories in this study were not specifically trained for this purpose but relied on their skills as midwives and doctors during routine clinical care. Third, due to the retrospective nature of our study, we are unable to determine the proportion of fetuses that were not systematically examined in the antepartum and intrapartum stillbirth categories. This limit, for example, the extent, to which our findings of a relatively non-existence of congenital infection as a cause of stillbirth in our setting may be interpreted. However, we consider that our study is a critical initial step in unravelling broad categories of causes of stillbirths in a resource-limited setting like ours. Lastly, the WHO ICD-PM classification system provides clinically relevant and easy-to-use categorisation of the causes of stillbirths including time of stillbirth (ante- or intrapartum), assigns fetal and/or maternal contributory conditions. However, other classification systems that examine care-related factors alongside fetal and maternal contributory factors exist and may enhance the identification of prevention priorities at the macro- and meso-levels of the health system [10].

There were two peaks in stillbirth (in 2012 and 2015) in our study. Although Gombe (our study setting) is in the northeast of Nigeria, it was considered a “non-conflict state”, i.e. a State with a low intensity of *Boko-Haram* attacks [12]. This unique characteristic made FTHG (the study setting) relatively safer and functional and it served as the major tertiary-level referral centre for maternal health services from surrounding States in northeast Nigeria. Thus, there might have been an increased migration of people and referral of women with complicated pregnancies from “conflict affected” (i.e. States with high intensity of *Boko Haram* attacks) states in northeast Nigeria to Gombe in 2012 and 2015, which coincided with peaks in stillbirth rates. However, the data on the intensity of armed conflicts in northeast Nigeria between 2009 and 2018 did not suggest substantially higher intensity of *Boko Haram* attacks in 2012 and 2015 in the “conflict affected” areas [23].

Beginning from 2015, a slight annual reduction in the SBR of about 2% was observed, but this is considerably lower than the 4.2% reduction rate recommended to achieve the *ENAP* target of < 12 deaths per 1000 livebirths by 2030 [1]. At the current rate of decline, the 2030 target will not be met, and it will take decades at the present rate of progress before the average pregnant women has the same chance of having a live birth as does a woman nowadays in a high-income country [1]. A monumental shift in SBR reduction is therefore required to get on-track. One reason for the observed slow decline in the SBR may be the armed conflict affecting parts of northeast Nigeria which has negatively impacted the utilisation of health services such as ANC and facility-based delivery, and hampered programmatic efforts aimed at delivering improved health services [6]. Stillbirths are strongly linked to adverse social and economic determinants of health. In a region of Nigeria with an already significantly high burden of maternal, perinatal, infant and childhood mortality [24], failure to address the impact of the complex political, socioeconomic and security issues on health coverage will widen equity gaps between regions and make it impossible for Nigeria to make progress without leaving anyone behind.

Based on the time of death, M4 (maternal medical and surgical) conditions caused more antepartum death while M1 (disorders of placenta, cord and membranes) conditions resulted in more intrapartum deaths. This result differs from that of Aminu et al. [11] also based in west Africa, where M1 (disorders of placenta, cord and membranes) and M3 (intrapartum complications) conditions were the most frequent maternal causes of stillbirth in the antepartum and intrapartum periods respectively. A plausible explanation for our finding is that maternal medical conditions like hypertensive disorders in pregnancy, diabetes mellitus and anaemia are more likely to cause fetal demise before the onset of labour. In contrast, intrapartum complications such as abruptio placentae and cord prolapse (M1), and obstructed labour (M3) causes intrapartum death. Although the study by Aminu et al. was prospective, there were methodological similarities with our study in the assignment of cause and time of fetal death. Despite contact with a health system before, during and after pregnancy, many women and their newborns do not receive high quality care. Ensuring high quality ANC will enable early detection and treatment of maternal medical conditions. Immediate access to emergency obstetric services when complications occur is crucial to the survival of mother and child, and can provide effective interventions to prevent or treat placenta and intrapartum complications [11].

The predominant antepartum and intrapartum fetal causes of stillbirth were prematurity/growth disorders (A5/I6). This finding contrasts data from a previous study by Aminu et al. [11] where congenital infections (A2) and acute intrapartum events (I3) constituted the majority of antepartum and intrapartum causes respectively. In contrast to the ‘unidentified’ maternal conditions (M5) which constituted a small proportion of the maternal causes of stillbirth in our setting, ‘unspecified’ ante- and intrapartum fetal conditions (A6/I7) made up a significant proportion of fetal causes of stillbirth. We believe this is due to the lack of systematic fetal examination after the delivery of a stillborn [25]. As is the case in most sub-Saharan African settings, the routine examination of stillborn infants at the FTHG consists of weight measurement and gross general examination without an accurate estimation of gestational age. Furthermore, the non-performance of autopsies, histopathological examination of placenta tissues and serological studies, hinders the definitive diagnoses of congenital infections such as Syphilis and Malaria (A2 & I4) which may constitute a significant group in the aetiology of stillbirths [26]. Autopsies, placenta tissue histology and fetal serological studies are not routinely performed in many LMICs [25]. In their study on causes of stillbirths in South Africa, Madhi et al. [27] performed histologic analysis of placenta and fetal blood culture, and found congenital infections as responsible for 19% of stillbirths. Future prospective studies in our setting exploring the contribution of infection to stillbirths using methods similar to those deployed by Madhi et al. [27] in South Africa will be warranted to provide much needed aetiological information which is lacking but necessary to prevent future deaths, improve the quality of care and guide resource allocation.

## Conclusions

Our study provides trend data and useful insights on the causes of stillbirth in the northeast region of Nigeria. Unless present SBR trends change, equity gaps between regions in Nigeria, particularly conflict-stricken areas, continue to widen over time. Most stillbirths, especially intrapartum stillbirths, are preventable; however, accelerating progress to end preventable stillbirths requires improved data which in turn requires improvements in hospital and national information systems. Such data will strengthen accountability for evidenced-based effective interventions.

## Supplementary information

**Additional file 1: Table S1.** WHO ICD-PM stillbirth classification. **Table S2.** Summary of births, stillbirths, stillbirth rates and stillbirth cases retrieved per years at the Federal Teaching Hospital, Gombe, Nigeria, 2010–2018. **Table S3.** Summary of major clinical conditions under the ICD-PM sub-categories at the Federal Teaching Hospital, Gombe, Nigeria, 2010–2018.

## Data Availability

All data are available from the corresponding author on reasonable request.

## Abbreviations

ANC: Antenatal Care
ENAP: Every Newborn Action Plan
FTHG: Federal Teaching Hospital Gombe
GBD: Global Burden of Diseases
ICD- PM: International Classification of Disease (10th Revision)- Perinatal Mortality
LMICS: Low-and Middle-Income Countries
MDG: Millennium Development Goals
SBR: Stillbirth Rate
SDG: Sustainable Development Goals
UNICEF: United Nations Children’s Fund
WHO: World Health Organization
