# Supplementary material for: Applying the WHO ICD-PM classification system to stillbirths in a major referral Centre in Northeast Nigeria: a retrospective analysis from 2010-2018
Source: BMC Pregnancy Childbirth. 2020 Jul 1;20:383. doi: 10.1186/s12884-020-03059-8 (PMC7329521; doi:10.1186/s12884-020-03059-8)
Supplement: Supplementary file 1 — Additional file 1: Table S1. WHO ICD-PM stillbirth classification. Table S2. Summary of births, stillbirths, stillbirth rates and stillbirth cases retrieved per years at the Federal Teaching Hospital, Gombe, Nigeria, 2010–2018. Table S3. Summary of major clinical conditions under the ICD-PM sub-categories at the Federal Teaching Hospital, Gombe, Nigeria, 2010–2018. [file 12884_2020_3059_MOESM1_ESM.docx]

**Supplementary Table 1: WHO ICD-PM stillbirth classification***

| **ICD-PM stillbirth group** | **Sub-group categories** | **Main conditions in the groups** |
| --- | --- | --- |
| **Antepartum fetal conditions** (**A**) | **A1** - Congenital malformations, deformations and chromosomal abnormalities | Anencephaly, spina bifida, congenital hydrocephalus, congenital malformations of limb(s) etc. |
|  | A2 – Infections | Congenital syphilis, congenital viral hepatitis, congenital rubella syndrome, congenital falciparum malaria etc. |
|  | **A3** - Antepartum hypoxia | Intrauterine hypoxia |
|  | **A4** - Other specified antepartum disorder | Fetal blood loss from vasa praevia, ruptured cord, twin-twin transfusion syndrome, rhesus isoimmunization etc. |
|  | **A5** - Disorders related to fetal growth | Slow fetal growth and fetal malnutrition, prematurity, post-term infant, macrosomia etc. |
|  | **A6** - Antepartum death of unspecified cause | Fetal death of unspecified cause |
| **Intrapartum fetal conditions** (**I**) | **I1** - Congenital malformations, deformations and chromosomal abnormalities | Anencephaly, spina bifida, congenital hydrocephalus, congenital malformations of limb(s) etc. |
|  | **I2** - Birth trauma | Subdural haemorrhage, cerebral haemorrhage, spinal cord injury, skull fracture etc. |
|  | **I3** - Acute intrapartum event | Intrauterine hypoxia |
|  | **I4** – Infection | Congenital syphilis, congenital viral hepatitis, congenital rubella syndrome, congenital falciparum malaria etc. |
|  | **I5** - Other specified intrapartum disorder | Fetal blood loss from vasa praevia, ruptured cord, twin-twin transfusion syndrome, rhesus isoimmunization etc. |
|  | **I6** - Disorders related to fetal growth | Slow fetal growth and fetal malnutrition, prematurity, post-term infant, macrosomia etc. |
|  | **I7** - Intrapartum death of unspecified cause | Fetal death of unspecified cause |
| **Maternal conditions** (**M**) | **M1** - Complications of placenta, cord and membranes | placenta praevia, placenta abruption, cord prolapse, chorioamnionitis etc. |
|  | **M2** - Maternal complications of pregnancy | oligohydramnios/polyhydramnios, multiple pregnancy, preterm rupture of membranes etc. |
|  | **M3** - Other complications of labour and delivery | breech delivery and extraction, obstructed labour, instrumental delivery, caesarean section etc. |
|  | **M4** - Maternal medical and surgical conditions | Hypertensive disorders, maternal diabetes, infectious disease, nutritional disorders, maternal injury etc. |
|  | **M5** - No maternal condition | no maternal condition identified (healthy mother) |

*Adapted from the WHO ICD-PM classification

**Supplementary Table 2:** **Summary of births, stillbirths, stillbirth rates and stillbirth cases retrieved per years at the Federal Teaching Hospital, Gombe, Nigeria, 2010-2018.**

| **Years** | **Births (n)** | **Stillbirths (n)** | **Stillbirth rate per 1000 births (95% CI)** | **Stillbirths retrieved (n)** | **Retrieval rate (%)** |
| --- | --- | --- | --- | --- | --- |
| 2010 | 2090 | 88 | 42 (34, 51) | 57 | 65 |
| 2011 | 3225 | 188 | 58 (50, 66) | 129 | 69 |
| 2012 | 3073 | 191 | 62 (53, 71) | 125 | 65 |
| 2013 | 2520 | 132 | 52 (44, 61) | 97 | 73 |
| 2014 | 1401 | 85 | 61 (50, 73) | 53 | 62 |
| 2015 | 1936 | 126 | 65 (55, 76) | 76 | 60 |
| 2016 | 2602 | 135 | 52 (43, 60) | 75 | 56 |
| 2017 | 2437 | 125 | 51 (43, 60) | 79 | 63 |
| 2018 | 2178 | 107 | 49 (40,58) | 69 | 64 |
| **Overall** | **N=21,462** | **N=1,177** | **55 (52, 58)** | **N=760** | **65** |

**Supplementary Table 3: Summary of major clinical conditions under the ICD-PM sub-categories at the Federal Teaching Hospital, Gombe, Nigeria, 2010-2018.**

| **ICD-PM group** | **ICD-PM sub-categories** | **Major clinical conditions in sub-category** |
| --- | --- | --- |
| **Antepartum fetal conditions** **(A)** | **A1**: ***Congenital malformations, and chromosomal abnormalities: n (%)*** | |
|  |  | Neural tube defects: 6 (54.5) |
|  |  | Other congenital anomalies: 5 (45.5) |
|  | ***Total*** | ***11 (100)*** |
|  | **A3**: ***Antepartum hypoxia*** | |
|  |  | Antepartum asphyxia: 35 (100) |
|  | ***Total*** | ***35 (100)*** |
|  | **A5**: ***Disorder related to fetal growth: n (%)*** | |
|  |  | Prematurity: 180 (87.4) |
|  |  | Fetal Macrosomia: 16 (7.8) |
|  |  | Others: 10 (4.8) |
|  | ***Total*** | ***206 (100)*** |
| **Intrapartum fetal conditions (I)** | **I1**: ***Congenital malformations, and chromosomal abnormalities: n (%)*** | |
|  |  | Neural tube defects: 11 (55) |
|  |  | Other congenital anomalies: 9 (45) |
|  | ***Total*** | ***20 (100)*** |
|  | **I3**: ***Acute intrapartum event: n (%)*** | |
|  |  | Intrapartum asphyxia: 31 (75) |
|  |  | Others: 10 (25) |
|  | ***Total*** | ***41 (100)*** |
|  | **I6**: ***Disorder related to fetal growth: n (%)*** | |
|  |  | Prematurity: 156 (88.6) |
|  |  | Fetal Macrosomia: 15 (8.5) |
|  |  | Others: 5 (2.9) |
|  | ***Total*** | ***176 (100)*** |
|  | **M1*: Complications of placenta, cord, etc.: n (%)*** | |
|  |  | Placenta abruption: 166 (78.7) |
|  |  | Cord prolapse: 20 (9.5) |
|  |  | Placenta previa: 19 (9) |
|  |  | Chorioamnionitis: 6 (2.8) |
|  | ***Total*** | ***211 (100)*** |
|  | **M2**: ***Maternal complications of pregnancy: n (%)*** | |
|  |  | Multiple gestation: 25 (53.2) |
|  |  | Poly/Oligohydramnios: 12 (25.5) |
|  |  | Premature rupture of membranes: 10 (21.5) |
|  | ***Total*** | ***47 (100)*** |
| **M3**: ***other complications of labour & delivery: n (%)*** | | |
| **Maternal conditions (M)** |  | Obstructed labour: 73 (65.2) |
|  |  | Ruptured uterus: 32 (28.6) |
|  |  | Others: 7 (6.2) |
|  | ***Total*** | ***112 (100)*** |
|  | **M4:** ***Maternal medical & surgical conditions: n (%)*** | |
|  |  | Hypertensive disease in pregnancy*: 218 (69) |
|  |  | Maternal severe anaemia: 70 (22) |
|  |  | Diabetes Mellitus: 22 (6.8) |
|  |  | Others: 9 (2.2) |
|  | ***Total*** | ***319 (100)*** |

***Note:*** *ICD-PM categories A2/I4 (infection), A4 (Other specified antepartum disorder), I2 (birth trauma) and I5 (Other specified intrapartum disorder) are not included in the table because these subgroups had values of one or none. *Hypertensive disease in pregnancy is a spectrum and included maternal chronic hypertension, pregnancy induced hypertension (PIH), preeclampsia and eclampsia*.
